# Efficacy of granulocyte-macrophage colony-stimulating factor combined with metronomic paclitaxel in the treatment of Lewis lung carcinoma transplanted in mice

**DOI:** 10.18632/oncotarget.23530

**Published:** 2017-12-21

**Authors:** Nengping Zhu, Rongsheng Qin, Qin Zhang, Shaozhi Fu, Shanshan Liu, Yue Chen, Juan Fan, Yunwei Han

**Affiliations:** ^1^ Department of Oncology, The Affiliated Hospital of Southwest Medical University, Luzhou, 646000, China; ^2^ Department of Gastroenterology, First People's Hospital of Liangshan Yi Autonomous Prefecture, Xichang, 615000, China; ^3^ Department of Nuclear Medicine, The Affiliated Hospital of Southwest Medical University, Luzhou, 646000, China

**Keywords:** lung cancer, paclitaxel, metronomic chemotherapy, GM-CSF, dendritic cell

## Abstract

Metronomic chemotherapy in combination with immunotherapy is an attractive approach in cancer therapy. The purpose of the present study was to investigate the anti-tumor effect of granulocyte-macrophage colony-stimulating factor (GM-CSF) in combination with metronomic paclitaxel (MET PTX) on Lewis lung carcinoma transplanted in mice. In the present study, tumor-bearing mice survival time and tumor growth were monitored. The day after the end of the treatment, white blood cells were counted, and the number and maturation of dendritic cell were determined by flow cytometry. Besides, microvessel density and tumor cell proliferation were determined by immunohistochemistry, while apoptosis was determined by TUNEL (Terminal deoxynucleotidyl transferase-mediated nick end labeling) assay. Micro ^18^F-FDG PET/CT (^18^F-Fluorodeoxyglucose positron emission tomography/computed tomography) was used to obtain SUVmax values. White blood cells reduction was not observed in the mice treated with GM-CSF combined with MET PTX. Moreover, GM-CSF combined with MET PTX further reduced proliferation and microvessel density, promoted tumor apoptosis, increased the dendritic cells number and induced their maturation, with concomitant delay in tumor growth and improved survival. Taken together, GM-CSF combined with MET PTX exerted a synergistic anti-tumor effect against lung cancer in a mouse model through an antiangiogenic activity and inducing dendritic cells maturation without exerting pronounced adverse effects. Hence, combined metronomic chemotherapy and immunotherapy could be a potential strategy for the treatment of patients with advanced lung cancer.

## INTRODUCTION

Lung cancer is the leading cause of cancer related deaths in China with increasing incidence and mortality [1]. The most common lung cancer form (83%) is non-small-cell lung cancer (NSCLC). Because NSCLC at early stage lacks specific symptoms, 57% of these patients are diagnosed at an advanced stage [2]. Conventional chemotherapy remains the standard treatment for the non-selective advanced NSCLC, which means a cyclic administration of chemotherapeutic drugs in a maximum tolerated dose (MTD) to exert its anti-tumor effect [3]. MTD chemotherapy can systemically kill most tumor cells, although simultaneously destroying non-tumor cells, leading to immunosuppression and other dose-limiting side effects. Most patients with advanced NSCLC are not able to tolerate the adverse effects of MTD chemotherapy, resulting in poor prognosis. Thus, there is an urgent need for more effective and tolerable treatments to improve the quality of life and prolong the survival time.

Metronomic chemotherapy is an emerging strategy to optimize cancer therapy, which is a therapeutic regimen consisting of frequent administration of antineoplastic drugs at a relatively low dose without a long rest period [4]. It has been demonstrated that chemotherapeutic drugs administrated at low dose are superior to those at MTD in terms of adverse reactions and drug resistance [5]. The anti-tumor mechanism of metronomic chemotherapy consists of an immunomodulatory effect besides the antiangiogenic effect [6]. Such immunomodulatory effect was enhanced in animal models by the combination with immunotherapy. In fact, preclinical models showed that the association of metronomic chemotherapy with immunotherapy such as cancer vaccine or adoptive cell therapy significantly inhibited tumor growth in tumor-bearing mice [7–10]. The reduced side effects and extended survival time were associated with the combined regimens [7, 8, 10]. These studies suggested the benefits of metronomic chemotherapy combined with immunotherapy in the treatment of this type of cancer. However, the complexity of obtaining a cancer vaccine or adoptive cells greatly limits the clinical application of these combined therapies. Thus, a simple and convenient immunotherapy is needed.

Dendritic cells can prime antigen-specific immune responses through acquiring and processing tumor antigens to lymphocytes [11]. Dendritic cells have a pivotal role in immunity development, indicating that strategies to enhance their function (such as their recruitment, activation or maturation) may enhance the anti-tumor effect. Granulocyte-macrophage colony-stimulating factor (GM-CSF) is an important growth and differentiation factor for dendritic cells [12], which is used as an immune adjuvant investigated in many tumor vaccine trials. These studies are devoted to prepare a GM-CSF-secreting tumor vaccine using genetic engineering, GM-CSF-surface-modified tumor vaccine using GM-CSF to cultivate tumor cells, and dendritic cell tumor vaccine using GM-CSF to cultivate dendritic cell [13–15]. However, a recent study found that GM-CSF directly administered through subcutaneous injection combined with radiotherapy can produce an anti-tumor immunity in some NSCLC patients [16]. Indeed, local radiotherapy can induce antigen exposure from dying tumor cells and recruit immune cells in synergy with GM-CSF to enhance the anti-tumor effect.

Several recent studies have found that metronomic paclitaxel (MET PTX) may increase tumor antigen exposure and induce dendritic cells maturation, suggesting that MET PTX may improve the outcome of immunotherapy [17, 18]. Since MET PTX and radiotherapy have the same function, we wondered whether MET PTX anti-tumor effect could be enhanced by GM-CSF just like radiotherapy. We assumed that GM-CSF could augment dendritic cells function acting synergistically with MET PTX to provide enhanced anti-tumor responses. Thus, this study was performed to verify this hypothesis in a lung cancer model, with the aim to explore a novel treatment.

## RESULTS

### GM-CSF combined with MET PTX significantly inhibited tumor growth in tumor-bearing mice

**Figure 1:**
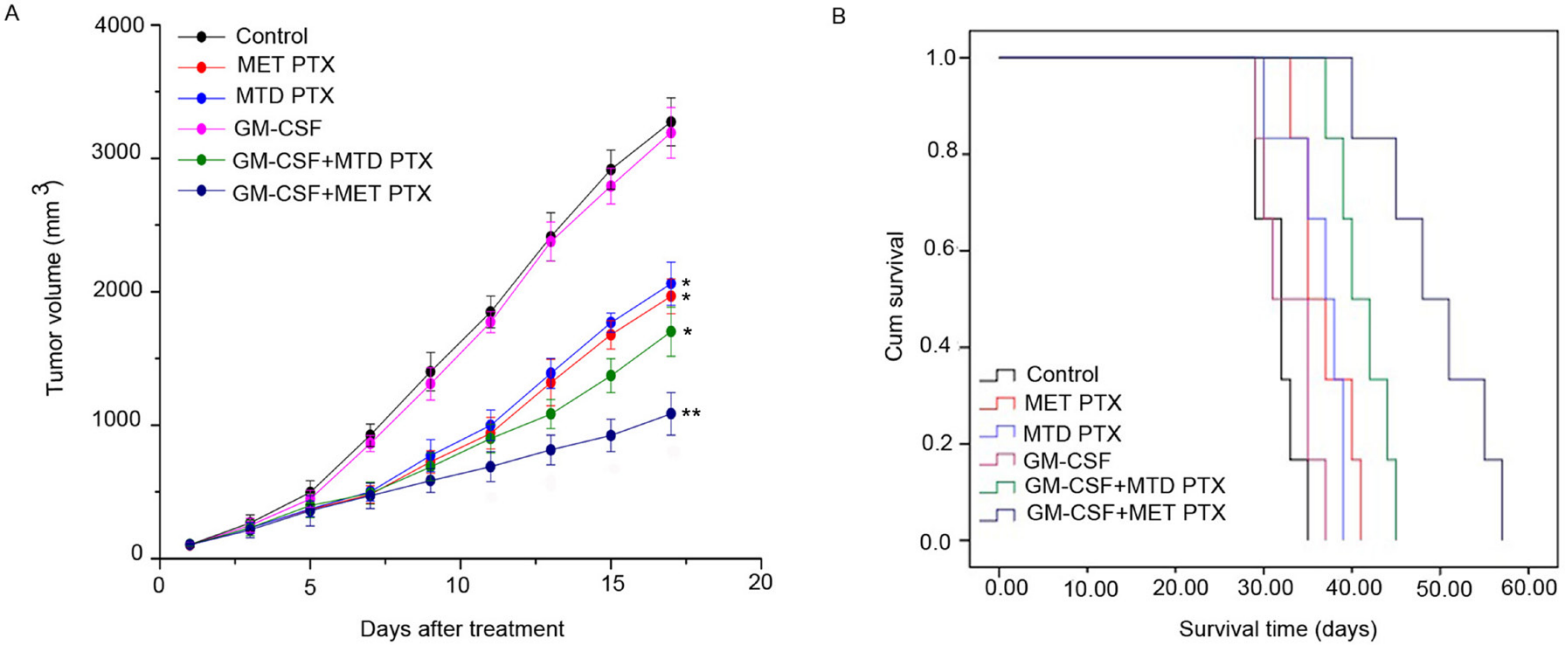
Tumor size and survival time of C57BL/6 mice (**A**) Tumor growth curves of each mice group. Data are expressed as mean ± SD (*n* = 6 per group). ^*^P < 0.05 vs control group; ^**^P < 0.05 vs all the other groups. (**B**) Kaplan-Meier survival curves of each group (*n* = 6 per group).

The average transplanted tumor volume in different groups is shown in Figure 1A. The tumor in the control group and GM-CSF group grew faster than that in the other groups, and the difference between the two groups was not significant, indicating that treatment with GM-CSF alone could not effectively inhibit the growth of transplanted tumors *in vivo*. Mice treated with MET PTX or MTD PTX could delay the tumor growth compared to the control group (*P* < 0.05), although no significant difference was observed between the two groups. Mice receiving GM-CSF plus MTD PTX also showed tumor growth inhibition compared to the control group (*P*<0.05). However, GM-CSF combined with MET PTX group showed the smallest tumor volume compared to the other groups (all *P* < 0.05). The tumor volume inhibition rate in the MET group, MTD group, GM-CSF+MTD PTX group and GM-CSF+MET PTX group were 39.91%, 37.04%, 48.03% and 66.85%, respectively. These results suggested that GM-CSF combined with MET PTX had the most effective anti-tumor effect among all groups.

### GM-CSF in combination with MET PTX prolonged survival time of tumor-bearing mice

Survival analysis of tumor-bearing mice was performed and Kaplan-Meier survival plots were generated (Figure 1B). GM-CSF had no effect on survival with a median survival time of 31 days compared to a median survival time of 32 days in control group. In contrast, mice in the MET PTX, MTD PTX and GM-CSF+MTD PTX groups survived a longer period, with a median survival time of 35, 37, 40 days, respectively. Most importantly, mice treated with GM-CSF combined with MET PTX exhibited a significant prolonged survival time with a median survival time of 48 days (*P* < 0.05, compared to the other groups). These data showed that GM-CSF combined with MET PTX could ameliorate the survival of tumor-bearing mice.

### GM-CSF combined with MET PTX significantly inhibited neovascularization and tumor cell proliferation

**Figure 2:**
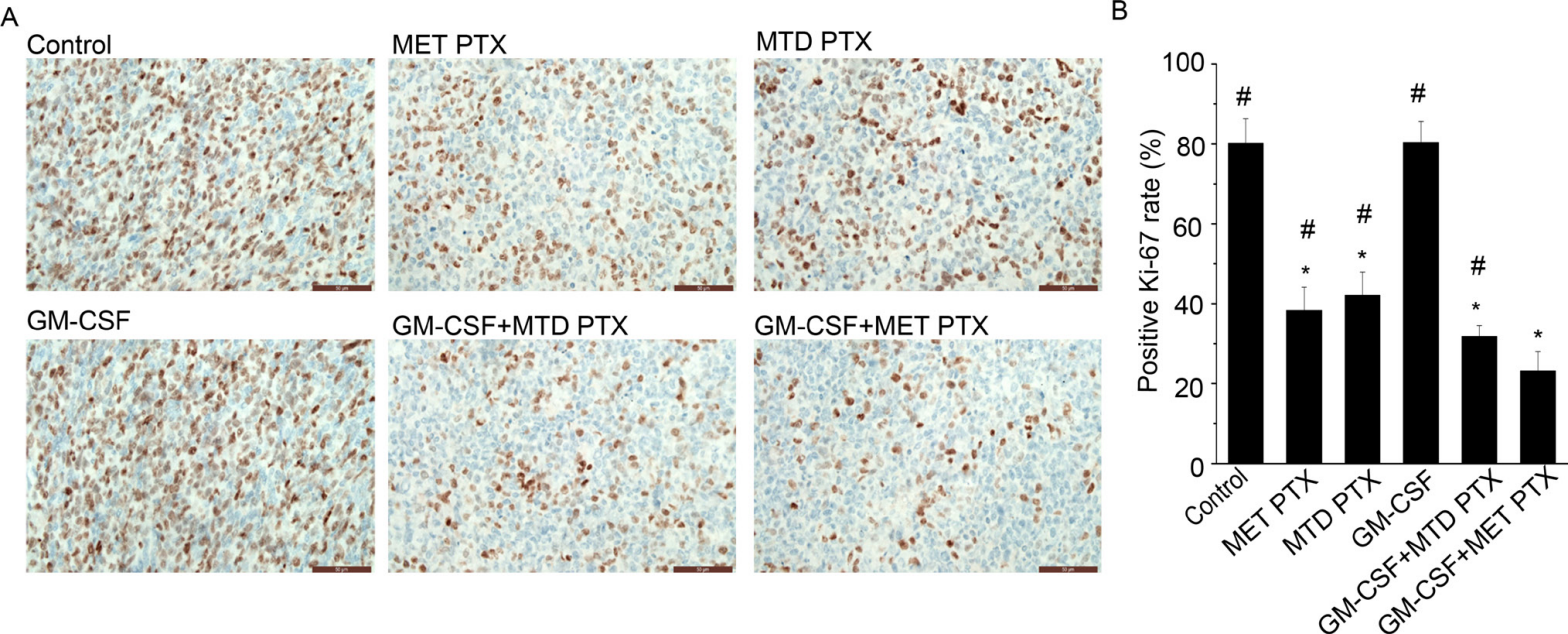
Ki-67 expression in transplanted tumors from different groups (**A**) Immunohistochemical images showing Ki-67 expression in transplanted tumors, scale bars = 50 μm (original magnification, ×400). (**B**) Histogram showing positive Ki-67 rate (%) in each group. Data are expressed as mean ± SD of the positive Ki-67 rate (%) in each group (*n* = 5 per group). ^*^P < 0.05 vs control group; ^#^*P* < 0.05 vs GM-CSF+ MET PTX group.

**Figure 3:**
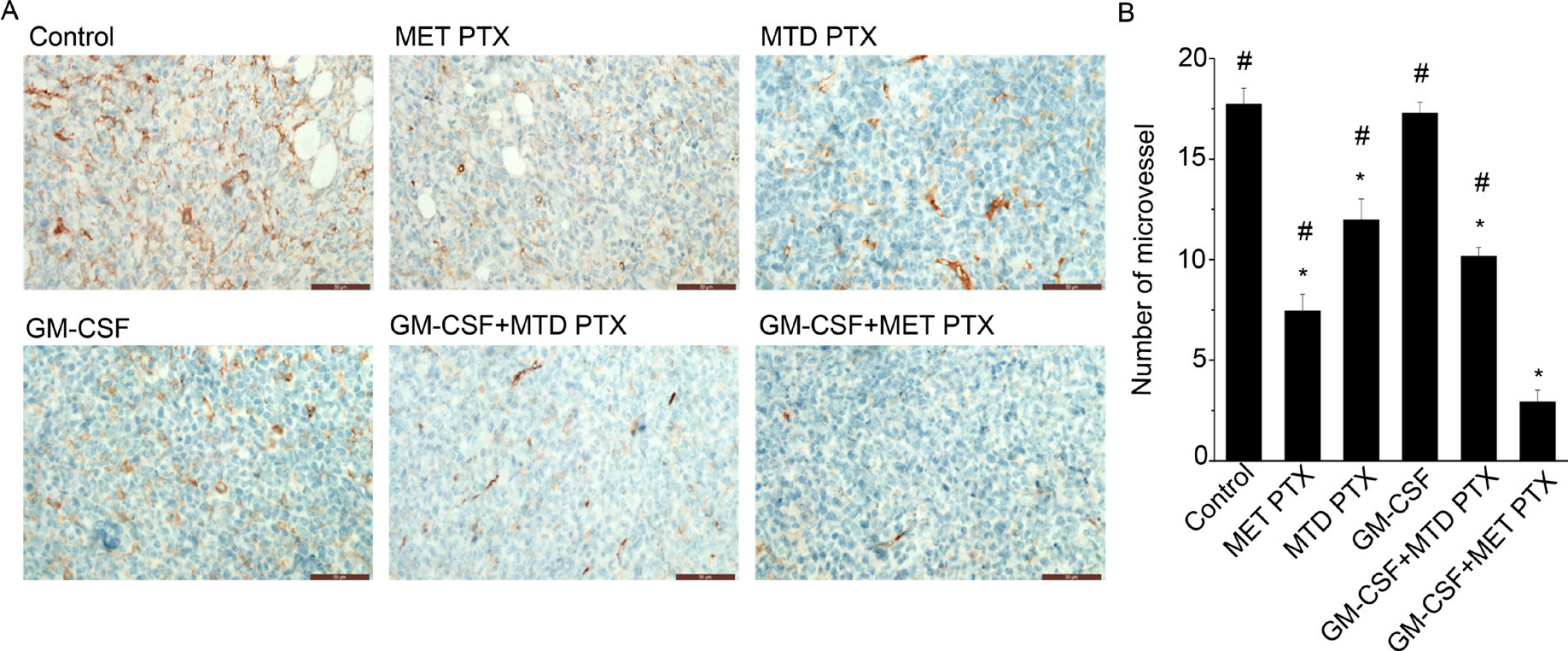
MVD in transplanted tumors from different groups was determined by immunofluorescence staining using an anti-CD31 antibody (**A**) Immunohistochemical images showing MVD changes in tumor tissue after different treatments, scale bars = 50 μm (original magnification, ×400). (**B**) Histogram showing the number of microvessel in each group. Data are expressed as mean ± SD of the number of microvessel in each group (*n* = 5 per group). ^*^P < 0.05 vs control group; ^#^*P* < 0.05 vs GM-CSF+ MET PTX group.

Tumor proliferation and microvessel density (MVD) in transplanted tumors were determined by immunofluorescence staining using antibody against Ki-67 (proliferation biomarker) and CD-31 (a specific endothelial cells biomarker), respectively. As shown in Figures 2 and 3, mice in control group and those in GM-CSF group showed similar proliferation index and MVD, and the difference between the two groups was not significant. Both MET PTX and MTD PTX group showed a reduced proliferation index and MVD value compared to control group (*P* < 0.05), and the MVD value was lower in MET PTX group than in MTD PTX group (*P* < 0.05 between the two groups). Mice treated with GM-CSF and MTD PTX also showed a reduced proliferation index and MVD value compared to control group (*P* < 0.05). However, GM-CSF+MET PTX group showed a significant decrease in proliferation index and MVD value, compared to MET PTX group, MTD PTX group or GM-CSF+MTD PTX group (all *P* < 0.05). These data indicated that MET PTX significantly reduced tumor-associated MVD and inhibited tumor cell proliferation to delay tumor growth. Taken together, GM-CSF+MET PTX could further inhibit tumor growth by exerting an anti-proliferative and antiangiogenic effect.

### GM-CSF combined with MET PTX significantly promoted apoptosis in tumor tissues

**Figure 4:**
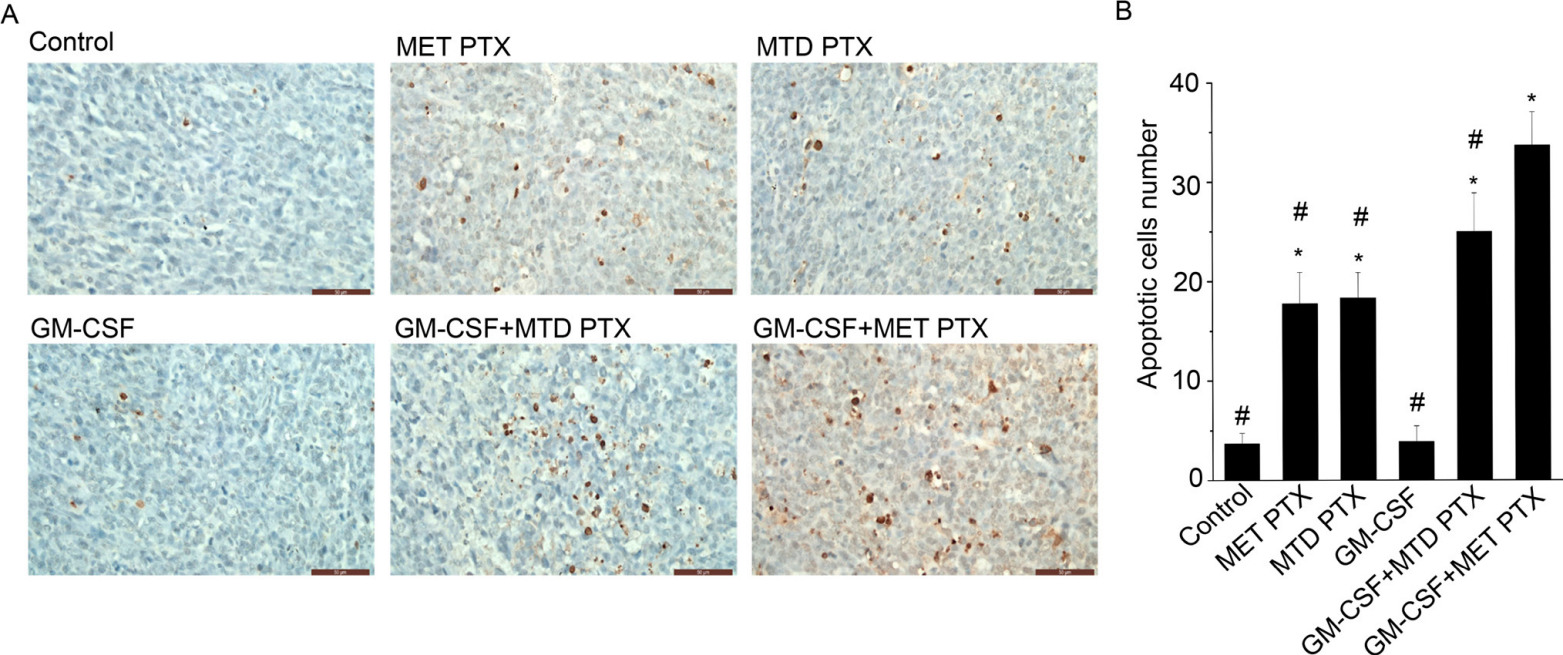
TUNEL assays in tumor tissue from different groups (**A**) Images showing cell stained in brown, scale bars = 50 μm (original magnification, ×400). (**B**) Histogram showing apoptotic cells number in each group. Data are expressed as mean ± SD of apoptotic cells number in each group (*n* = 5 per group). ^*^P < 0.05 vs control group, ^#^*P* < 0.05 vs GM-CSF+ MET PTX group.

Terminal deoxynucleotidyl transferase-mediated nick end labeling (TUNEL) assay was used to determine apoptosis in transplanted tumors under various regimens (Figure 4). Control (3.64 ± 1.13) and GM-CSF group (3.84 ± 1.60) showed less apoptotic cells than the other groups (*P* < 0.05), but no significant difference was observed between the two groups. Treatment with MET PTX (17.76 ± 3.15) or MTD PTX (18.32 ± 2.56) or GM-CSF+MTD PTX (24.96 ± 3.88) could effectively increase the number of apoptotic cells in tumor tissues compared to the control group (*P* < 0.05). However, GM-CSF+MET PTX group showed the highest number of apoptotic cells (33.64 ± 3.38, *P* < 0.05) compared to any other group. These results suggested that the combination of GM-CSF with MET PTX could effectively exert an anti-tumor effect through apoptosis induction.

### Micro ^18^F-FDG PET/CT

**Figure 5:**
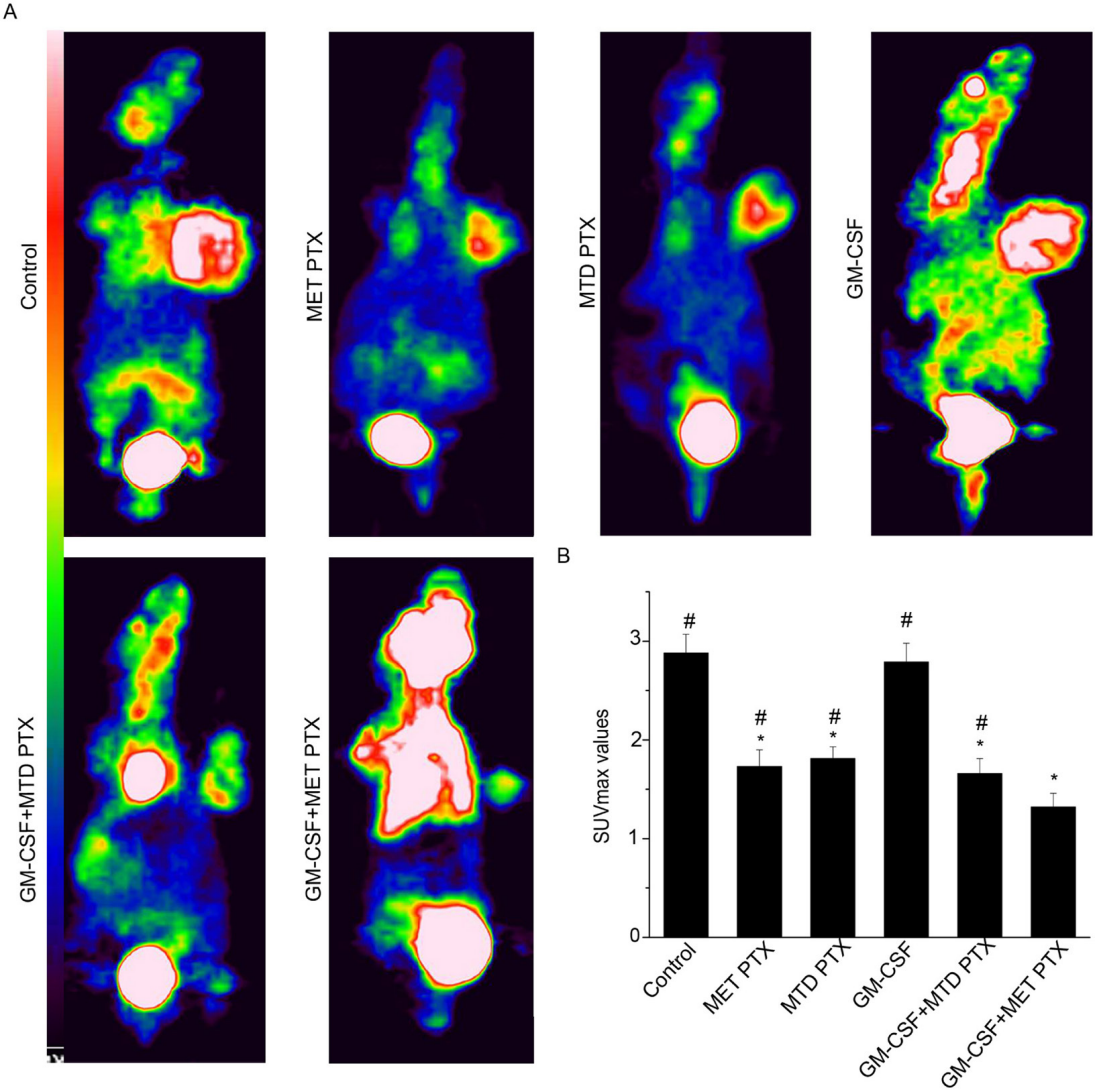
Micro ^18^F-FDG PET images of tumor bearing mice after different treatments (**A**) Representative ^18^F-FDG PET images from different groups. (**B**) SUVmax values of tumor bearing mice. Data are expressed as mean ± SD of SUVmax in each group (*n* = 6 per group). ^*^P < 0.05 vs control group; ^#^*P* < 0.05 vs GM-CSF+ MET PTX group.

^18^F-Fluorodeoxyglucose positron emission tomography/computed tomography (^18^F-FDG PET/CT) can be used to evaluate the treatment efficacy after different therapeutic regimens. Representative ^18^F-FDG PET/CT images and the maximal standardized uptake value (SUVmax) of tumor-bearing mice treated with various drug regimens are shown in Figure 5. SUVmax values of control, MET PTX, MTD PTX, GM-CSF and GM-CSF+MTD PTX group were 2.88 ± 0.19, 1.73 ± 0.17, 1.81 ± 0.12, 2.78 ± 0.19, 1.66 ± 0.15 respectively. Mice treated with PTX alone or combined with GM-CSF showed reduced SUVmax values compared to control group (*P* < 0.05). However, GM-CSF+MET PTX group SUVmax value (1.32 ± 0.14) was significantly lower than any other group (*P* < 0.05). These data further suggested that GM-CSF combined with MET PTX was effective in the treatment of Lewis lung carcinoma transplanted in mice.

### Side effects evaluation

In order to evaluate the safety of the different therapeutic regimens used, white blood cells (WBCs) number was calculated (Table 1). In addition, we analyzed the morphology of heart, liver, lung and kidney through hematoxylin and eosin (HE) staining. Tumor-bearing mice treated with MET PTX or MTD PTX had less WBCs than control group (*P* < 0.05), and a significant difference between the two groups was observed (*P* < 0.05). This result indicated that metronomic chemotherapy can reduce the degree of leukopenia. WBCs number in the GM-CSF+MET PTX group was similar to that of the control group, but no significant difference was observed between the two groups. HE stained sections of important targeted organs including heart, liver, lung and kidney of each group were observed, showing no pathological changes (Supplementary Figure 1). GM-CSF combined with MET PTX could effectively avoid WBC reduction without important targeted organs damage, indicating that this combination therapy could be well tolerated and safe.

**Table 1:** WBCs number change in different treatment groups and control group

Groups	*n*	WBC (×10^9^/L)
Control	6	12.18 ± 1.11
MET PTX	6	10.14 ± 0.99^*^
MTD PTX	6	8.37 ± 1.09^*^
GM-CSF	6	13.34 ± 1.44
GM-CSF+MTD PTX	6	11.85 ± 1.16
GM-CSF+MET PTX	6	12.87 ± 1.04

Data are expressed as mean ± SD. ^*^P < 0.05, compared to control group.

### GM-CSF combined with MET PTX increased dendritic cells number and induced their maturation

To investigate the effect of various regimens on dendritic cells, dendritic cells were harvested from spleens and tumor and analyzed by flow cytometry (Tables 2 and 3). Integrin CD11c is strongly expressed in dendritic cells, thus, it is used to mark dendritic cells [19]. Dendritic cells that express co-stimulatory molecules such as CD80 and CD86 could activate and differentiate naive T cells to induce immune response. Therefore, CD80 and CD86 are used as hallmarks of dendritic cell maturation [20]. GM-CSF alone could not effectively promote the percentage of dendritic cells and mature dendritic cells isolated from the spleen and the tumor compared to control group, and GM-CSF+ MTD PTX group revealed the same pattern. However, MET PTX could increase dendritic cells percentage both in the spleen and tumor and upregulate the expression of CD80 and CD86 co-stimulatory makers compared to control group (*P* < 0.05), while MTD PTX could reduce dendritic cells percentage and downregulate the expression of co-stimulatory makers CD80 and CD86 compared to control group (*P* < 0.05), with a significant difference between MET PTX and MTD PTX group (*P* < 0.05). Most importantly, the number of total and mature dendritic cells harvested from spleens and tumor in mice treated with GM-CSF plus MET PTX was significantly increased than that in mice treated with other regimens (*P* < 0.05). These results indicated that GM-CSF+MET PTX could induce the maturation and recruitment of dendritic cells in both spleens and tumor, thus exerting an anti-tumor immune effect and recruiting tumor derived dendritic cells. Therefore, tumor derived dendritic cells might be a good indicator for the efficacy of this therapeutic combination.

**Table 2:** CD11c, CD80 and CD86 positive cells percentage in the spleen

Groups	*n*	CD11c^+^	CD80^+^	CD86^+^
Control	6	3.37 ± 0.66	0.72 ± 0.05	0.63 ± 0.06
MET PTX	6	7.27 ± 0.79^*^	1.85 ± 0.08^*^	1.91 ± 0.09^*^
MTD PTX	6	2.67 ± 0.51^*^	0.42 ± 0.06^*^	0.45 ± 0.13^*^
GM-CSF	6	3.24 ± 0.38	0.75 ± 0.08	0.59 ± 0.10
GM-CSF+ MTD PTX	6	3.55 ± 0.56	0.79 ± 0.07	0.72 ± 0.08
GM-CSF+ MET PTX	6	10.07 ± 0.53^**^	3.40 ± 0.12^**^	3.75 ± 0.12^**^

Data are expressed as mean % ± SD. ^*^P < 0.05, compared to control group. ^**^P < 0.05, compared to all groups.

**Table 3:** CD11c, CD80 and CD86 positive cells percentage in the tumor

Groups	*n*	CD11c^+^	CD80^+^	CD86^+^
Control	6	4.60 ± 0.44	1.01 ± 0.09	0.96 ± 0.07
MET PTX	6	8.88 ± 0.63^*^	3.18 ± 0.16^*^	3.43 ± 0.22^*^
MTD PTX	6	3.35 ± 0.30^*^	0.52 ± 0.07^*^	0.61 ± 0.07^*^
GM-CSF	6	4.86 ± 0.55	0.98 ± 0.08	0.97 ± 0.10
GM-CSF+ MTD PTX	6	4.58 ± 0.47	1.05 ± 0.09	1.05 ± 0.10
GM-CSF+ MET PTX	6	12.26 ± 0.55^**^	6.30 ± 0.22^**^	6.68 ± 0.13^**^

Data are expressed as mean % ± SD. ^*^P < 0.05, compared to control group. ^**^P < 0.05, compared to all groups.

## DISCUSSION

In the present study, we evaluated the hypothesis that GM-CSF combined with MET PTX might provide an anti-tumor effect dramatically enhanced over GM-CSF or MET PTX alone and superior than GM-CSF combined with MTD PTX. Most importantly, this study highlighted the advantage of using GM-CSF as an immunotherapeutic agent, especially because GM-CSF is easier to obtain and use than other immunotherapeutic treatments in clinical practice. Thus, GM-CSF combined with MET PTX could be favorably considered and many patients may benefit from its effects.

One of the most common adverse effects of conventional chemotherapy is myelo-suppression, which can lead to severe infections and increase the duration of hospitalization [21], thus WBCs number was assayed to evaluate the safety of various regimens. In our study, although treatment with MET PTX or MTD PTX could both result in a leukocyte reduction compared to the control group, treatment with MET PTX could reduce the severity of leukopenia compared to that due to MTD PTX, confirming that metronomic chemotherapy could minimize the adverse effects of conventional chemotherapy [5]. What is more important, WBC reduction was absent when GM-CSF combined with MET PTX was used. GM-CSF is well-known as a hemopoietic growth factor which has been used in patients with myelo-suppression due to chemotherapy, thereby potentially allowing tumor-bearing mice to recover from WBC reduction caused by metronomic chemotherapy. Moreover, there no pathological changes were observed in important targeted organs (heart, liver, lung and kidney). Taken together, GM-CSF combined with MET PTX resulted to be a well-tolerated and safe treatment, potentially beneficial especially in old age patients and patients with poor performance status.

Angiogenesis plays a vital role in tumor growth and metastasis, and antiangiogenic therapy represents a potential cancer treatment strategy [22]. An increased MVD is often detected in tumor tissue with a high level of angiogenesis and correlated with a poor prognosis [23]. Here we found that GM-CSF combined with MET PTX led to an enhanced antiangiogenic effect as compared to other regimens including GM-CSF alone, MET PTX alone, MTD PTX alone and GM-CSF combined with MTD PTX. Growing evidence suggests that metronomic administrated chemotherapeutic agents were more active against tumor-associated endothelial cells in comparison to normal cells or cancer cells [24]. In addition, metronomic chemotherapy without long rest periods prevents recovery of the damaged tumor vasculature. Furthermore, GM-CSF can induce the expression of soluble vascular endothelial growth factor receptor-1 (sVEGFR1) by monocytes and inhibit angiogenesis *in vivo* [25, 26]. Thus GM-CSF and MET PTX combined treatment could maximize the antiangiogenic effect, leading to an increased number of apoptotic cells and necrosis. This in turn might contribute to the tumor antigens exposure from the dying tumor cells, thus eliciting immune responses [27].

Conventional chemotherapy is known to be associated with immunosuppression, thus it is not usually considered in combination with immunotherapy. Recent studies found that metronomic chemotherapy combined with immunotherapy has stronger anti-tumor effect than either metronomic chemotherapy or immunotherapy alone in animal models [7, 9, 27]. Zhong *et al.* demonstrated that MET PTX could enhance dendritic cells maturation and function [9]. GM-CSF is an important growth and differentiation cytokine for dendritic cells. Thus, we analyzed dendritic cells in spleen and tumor tissues to find the possible mechanism of action of MET PTX and GM-CSF combination. In our research, mice treated with GM-CSF alone could not effectively show a delay in tumor growth and enhance the function of dendritic cell compared to the control group. One explanation is that in the immunosuppressive environment created by tumor cells, treatment with GM-CSF alone could not elicit immune response, indicating that further treatment might be require using supplemental agents. Zhong *et al.* found that MET PTX combined with dendritic cell vaccine had an enhanced anti-tumor effect and could induce anti-tumor immunity in lung carcinoma model [9]. Consistent with this study, we found that GM-CSF in combination with MET PTX dramatically increased the number of dendritic cells compared to MET PTX alone or GM-CSF+MTD PTX. Furthermore, GM-CSF+MET PTX group expressed higher levels of the maturation markers CD80 and CD86 compared to any other group. These results indicated that GM-CSF in combination with MET PTX could improve the number and function of dendritic cell isolated from spleen and tumor tissues in tumor-bearing mice, which was associated with the mechanism of anti-tumor immune effect. Most importantly, GM-CSF combined with MET PTX increased infiltration of dendritic cells in tumor tissue, equivalent to intratumoral administrated dendritic cell vaccine. This is a promising method to construct an *in-situ* anti-tumor vaccine. However, further studies should be performed to explore the mechanism of action of this combined therapy.

To further prove the anti-tumor effect of GM-CSF combined with MET PTX on transplanted Lewis lung carcinoma, micro ^18^F-FDG PET/CT scans were used. Micro ^18^F-FDG PET/CT scan is a non-invasive test, which can assess the early tumor response by SUVmax values analysis. SUVmax values can be used as a prognostic maker, which is roughly negatively associated with prognosis [28, 29]. Our data showed that lower SUVmax values occurred in the tumors treated with MET PTX or treated with MTD PTX. However, mice treated with GM-CSF+MET PTX showed the lowest SUVmax values, suggesting that GM-CSF combined with MET PTX had effective anti-tumor ability and ^18^F-FDG PET/CT might be used to evaluate it.

In summary, our data demonstrated that GM-CSF combined with MET PTX exerted a synergistic anti-tumor effect in a lung cancer mouse model without pronounced adverse effects. This enhanced anti-tumor effect was exerted by limiting angiogenesis, inducing tumor cell apoptosis and inducing dendritic cells maturation. Hence, GM-CSF combined with MET PTX might become an effective treatment modality for advanced NSCLC. However, an evaluation of such combination regimen in other cancer models is recommended to further test its efficacy.

## MATERIALS AND METHODS

### Animals, cell line and transplanted tumor model

Female C57BL/6 mice 6–8 weeks old, weighing 20–22 g were purchased from Chongqing Tengxin biotechnology Co.Ltd (Chongqing, China). Mice were kept in a specific pathogen-free environment with steady temperature (24 ± 2°C), proper humidity (50 ± 10%) and 12 h light-dark cycles. Food, dietary supplements and water were provided ad libitum. The project was approved by the Institutional Animal Care and Treatment Committee of Southwest Medical University (Luzhou, China).

Lewis lung carcinoma cell line was provided by the laboratory of the oncology department of the Affiliated Hospital of Southwest Medical University (Luzhou, China). Transplanted tumor model was established through subcutaneous injection of Lewis lung carcinoma cells (2 × 10^6^ cells in 200 μL PBS) in the mice right armpit.

### Chemicals

PTX (30 mg/5 mL) was supplied by the Affiliated Hospital of Southwest Medical University (Luzhou, China). Recombinant Murine GM-CSF was purchased from PeproTech (Rocky Hill, USA).

### Experimental groups and design

When the tumor volume reached approximately 100 mm^3^, tumor-bearing mice were randomly divided into six groups (*n* = 18 per group): (1) control group: saline was administered once a day for a total of ten times at a dose of 0.1 ml/day; (2) MET PTX group: PTX was administered once every other day for a total of five times at a dose of 3 mg/kg/day; (3) MTD PTX group: PTX was administered on the first day of the experiment at a dose of 15 mg/kg; (4) GM-CSF group: GM-CSF was administered once a day for a total of ten times at a dose of 5 μg/kg/day; (5) GM-CSF+MTD PTX group: combination of (3) and (4); (6) GM-CSF+MET PTX group: combination of (2) and (4). PTX and saline were administered through intraperitoneal injection, while GM-CSF was administered through subcutaneous injection. Since the first day of drug administration tumor length (A) and width (B) were measured every other day. Tumor volume (V) was calculated using the formula V = (A × B^2^)/2.

After ten days of treatment, six mice of each group were randomly selected to remove eyeball and collect peripheral blood samples for WBCs count. Then, mice were sacrificed to collect tumor tissues and important target organs (heart, lung, spleen, liver and kidney) for further analysis. Tumor volume inhibition rate was calculated as follows: (1-volume of treatment group/volume of control group) × 100%. The remaining tumor-bearing mice were kept under observation until death occurred, and the survival time of each mouse was recorded.

### Histological analysis

Heart, lung, liver and kidney from each group were embedded in paraffin. Morphological changes were evaluated using HE staining under a microscope (Leica TE2000-S microscope, Japan).

### Immunohistochemistry and TUNEL assay

Tumor tissues were fixed with 10% neutral formaldehyde solution, embedded in paraffin and cut into 3–4 μm thick sections for immunohistochemical analysis. Ki-67 antibody (Bioworld technology Co. Ltd. Nanjing, China), CD-31 antibody (Bioworld technology Co. Ltd.) and *in situ* cell death detection kit (Roche, Mannheim, Germany) were used to detect Ki-67 expression, MVD and apoptosis, respectively. All staining steps were carried out according to the manufacturer's instructions. Images were taken by a microscope.

The proliferation index was quantified as the percentage of Ki-67-positively stained cells (brown cell nucleus), while apoptotic number was quantified as the number of TUNEL-positive cells (brown cell nucleus). MVD was evaluated according to the method described by Weidner *et al* [30]. MVD value was obtained as the mean number of CD-31-positive tubular structures in the five most vascular fields under high-power field (magnification ×400) per section.

### Micro ^18^F-FDG PET/CT

After the end of the treatment, six randomly selected mice of each group were fasted for 6 hours before PET/CT examination and next, they were anesthetized with 1% pentobarbital through intraperitoneal injection at the dose of 5 ml/kg. Approximately 30–40 minutes post intravenous injection of 100–200 μCi ^18^F-FDG, mice were placed in the Inveon micro PET-CT animal scanner (Siemens, Germany) to acquire PET/CT images. ^18^F-FDG uptake in tumor was assessed by calculating the SUV in a given region of interest. SUVmax was defined as the highest ^18^F-FDG uptake within a region of interest over tumor, which is the common used parameter and known as a prognostic maker.

### Dendritic cell characterization

Dendritic cells number was determined in the spleens and tumor tissues by flow cytometry (BD Biosciences, San Jose, CA). The sacrificed mice were soaked in 75% alcohol for 10 minutes and spleens and tumor mass of approximately 5 × 5 mm were collected. Spleens were mechanically dissociated in culture dish and dissociated cells were filtered through a 200-mesh sieve (Solarbio science & technology Co. Ltd. Beijing, China). Splenocytes single-cell suspensions were prepared from each mouse after red blood cells lysis. Tumor tissues were incubated in 1 mL trypsinization buffer at 37°C for 40 minutes after mechanical dissociation. Medium containing serum was added to terminate the tumor tissues digestion and dissociated cells were filtered through a 0.3-μm filter. Subsequently, cells were counted and stained using PE anti-mouse CD11c (1:100, Biolegend, San Diego, CA) to select dendritic cells, FITC anti-mouse CD80 (1:50, Biolegend) or APC anti-mouse CD86 (1:100, Biolegend) to distinguish mature dendritic cells from the immature ones. PE Armenian Hamster IgG (1:100, Biolegend), FITC Armenian Hamster IgG (1:50, Biolegend) and APC Rat IgG2a (1:100, Biolegend) were used as isotype controls.

### Statistical analysis

Statistical analysis was performed using SPSS 17.0 software (Chicago, IL, USA). All data were expressed as mean ± standard deviation (SD). Relative differences between multiple groups were evaluated using one-way analysis of variance (ANOVA). Survival analysis was performed using the log-rank test. *P* < 0.05 was considered statistically significant.

## SUPPLEMENTARY MATERIALS FIGURE


