# Supplementary material for: Efficacy of granulocyte-macrophage colony-stimulating factor combined with metronomic paclitaxel in the treatment of Lewis lung carcinoma transplanted in mice
Source: Oncotarget. 2017 Dec 21;9(4):4951–60. doi: 10.18632/oncotarget.23530 (PMC5797025; doi:10.18632/oncotarget.23530)
Supplement: Supplementary file 1 [file oncotarget-09-4951-s001.pdf]

Efficacy of granulocyte-macrophage colony-stimulating factor combined with metronomic paclitaxel in the treatment of Lewis lung carcinoma transplanted in mice

SUPPLEMENTARY MATERIALS

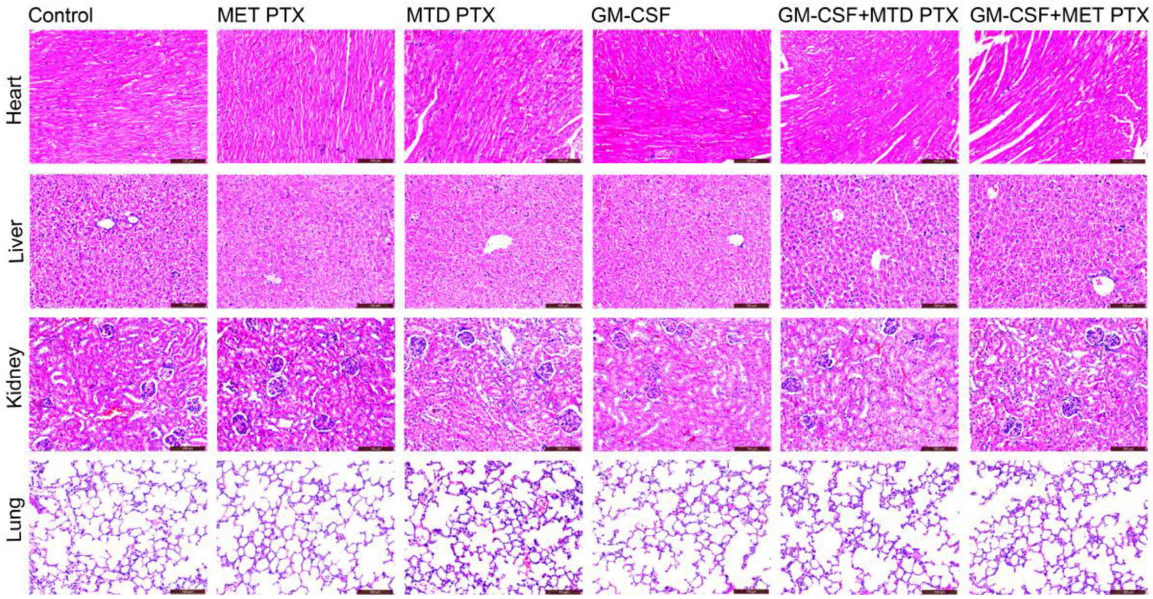

Supplementary Figure 1: Representative HE staining images of heart, liver, lung and kidney from different groups, scale bars = 100 µm (original magnification, ×200).
